# Dexmedetomidine: a real-world safety analysis based on FDA adverse event reporting system database

**DOI:** 10.3389/fphar.2024.1419196

**Published:** 2024-08-23

**Authors:** Yichun Shuai, Zhe Chen, Qiaoqian Wan, Jinzheng Wu, Xin Wang

**Affiliations:** ^1^ Department of Anesthesiology, Second Xiangya Hospital, Central South University, Changsha, Hunan, China; ^2^ Department of Thoracic Surgery, Second Xiangya Hospital, Central South University, Changsha, Hunan, China

**Keywords:** adverse event, Dexmedetomidine, FDA adverse event reporting system, real-world, safety

## Abstract

**Objective:**

Using the FDA adverse event reporting system (FAERS) database to analyze the safety profile of Dexmedetomidine and provide guidance for clinical application.

**Methods:**

Data from the FAERS database from the first quarter of 2004 to the third quarter of 2023 were collected. Reporting odds ratio (ROR), the proportional reporting ratio (PRR), and the Bayesian confidence propagation neural network (BCPNN) were employed to detect and assess adverse events associated with Dexmedetomidine.

**Results:**

A total of 1910 reports of Dexmedetomidine as the primary suspect drug were obtained. After screening, 892 preferred terms were obtained, including 52 new preferred terms not mentioned in the drug insert. The common adverse events of Dexmedetomidine include bradycardia, cardiac arrest, hypotension, diabetes insipidus, arteriospasm coronary and agitation. Notably, cardiac disorders exhibited the highest number of reports and the highest signal intensity in the system organ class. Among the new preferred terms, those with high signal intensity include transcranial electrical motor evoked potential monitoring abnormal, acute motor axonal neuropathy, trigemino-cardiac reflex, glossoptosis, floppy iris syndrome, phaeochromocytoma crisis, postresuscitation encephalopathy and diabetes insipidus.

**Conclusion:**

This study mined and evaluated adverse events associated with Dexmedetomidine and also identified new adverse events. This could help alert clinicians to new adverse events not mentioned in the drug inserts, reducing the risk of drug.

## 1 Introduction

Dexmedetomidine is a sedative medication. It is a very specific agonist for $\alpha_{2}$ adrenergic receptors (Keating, 2015). Its sedative effect is achieved by acting on the $\alpha_{2}$ adrenergic receptors in the locus coeruleus of the brainstem, which is the central hub for regulating wakefulness. This action prevents the neurons in the locus coeruleus from discharging and reduces the activity of the noradrenergic pathway. It also has analgesic properties, which can reduce the use of opioid. Dexmedetomidine has the ability to minimize respiratory depression, reduce sympathetic tone and mitigate the stress response induced by anesthesia and surgery, as well as diminish the occurrence of hypotension, delirium, tremors, and postoperative nausea and vomiting. Furthermore, patients treated with Dexmedetomidine were more prone to regain consciousness and exhibit cooperative and communicative behavior after awakening compared to other sedative medications (Chrysostomou and Schmitt, 2008; Keating, 2015; Grape et al., 2019). Therefore, Dexmedetomidine finds extensive application in providing sedation in intensive care units (ICU), emergency departments, regional and general anesthesia, and in various surgical, endoscopic, and radiologic procedures (Mantz et al., 2011; Afonso and Reis, 2012). With the widespread use of Dexmedetomidine in clinical settings, reports of associated adverse events (AEs) have increased. The most prevalent AEs associated with Dexmedetomidine include hypotension, hypertension, and bradycardia (Keating, 2015). In a meta-analysis investigating the use of Dexmedetomidine as a supplemental agent during general anesthesia for gynecologic surgery, it was observed that patients receiving perioperative Dexmedetomidine faced a notably increased risk of bradycardia (Hung et al., 2023). In another Meta-analysis, Dexmedetomidine showed a significant increase in the risk of bradycardia compared to propofol in ICU (Heybati et al., 2022). However, information on the safety of Dexmedetomidine has been documented primarily through clinical trials and meta-analyses, most of which documented cardiovascular-related adverse events. Furthermore, randomized controlled trials may fail to capture rare AEs due to short follow-up times, different criteria for study population selection, and limited sample sizes (Heybati et al., 2022). Therefore, it becomes crucial to collect safety data related to Dexmedetomidine in real-world settings.

The FAERS database is composed of AEs for drugs submitted by consumers, pharmacists, healthcare industry practitioners, and others. It is an open, spontaneous reporting system that records AEs occurring after a drug has been marketed and used by a wide range of people. The aim of this study was to analyze AEs related to Dexmedetomidine in the FAERS database and provide guidance for its clinical application.

## 2 Methods

### 2.1 Data source

**FIGURE 1:**
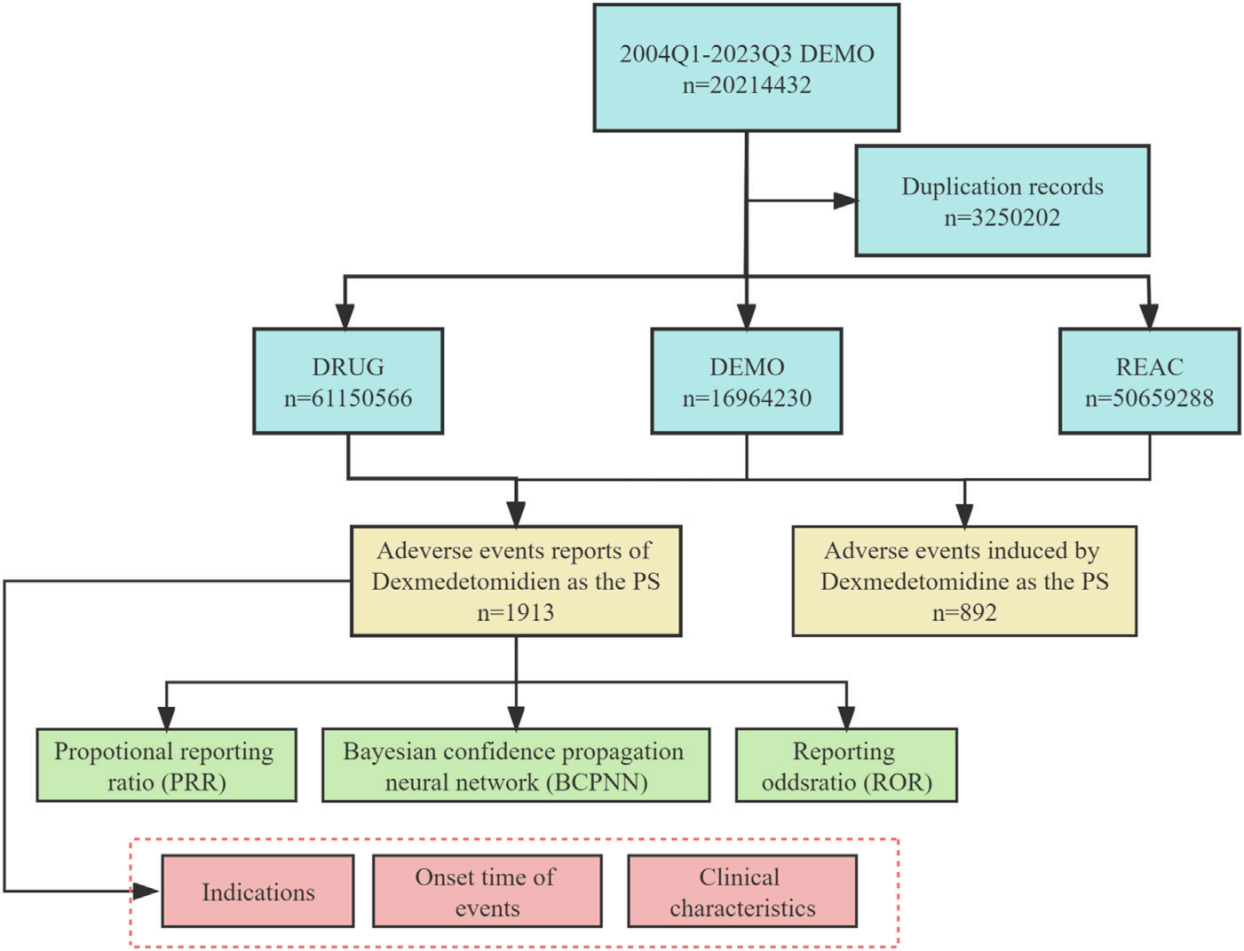
The flow diagram of selecting Dexmedetomidine-related AEs from FAERS database.

This study aims to assess the safety of Dexmedetomidine in real-world postmarketing setting. We collected adverse event reports with Dexmedetomidine as the primary suspect (PS) drug from the FAERS database, covering the period from the first quarter of 2004 to the third quarter of 2023. Data collection and cleaning were performed using R studio. The FAERS database generated 20,214,432 reports, and 32,502 duplicate reports were removed following FDA guidelines. Removal of duplicate reports refers to selecting the latest FDA_DT when CASEID and FDA_DT are the same in the DEMO table. Otherwise, choose the PRIMARYID with the greater value (Wang et al., 2023). All AEs in FAERS were categorized using preferred term (PT) and system organ class (SOC) using the Medical Dictionary for Regulatory Activities 20.0 (MedDRA 20.0) (Zhang et al., 2023). A total of 1913 adverse event reports associated with Dexmedetomidine were identified and 892 preferred terms (PTs) related to Dexmedetomidine were obtained in this study. Figure 1 illustrates the flowchart for selecting Dexmedetomidine-related AEs based on the FAERS database.

### 2.2 Statistical analysis

In our study, we used the reporting odds ratio (ROR), the proportional reporting ratio (PRR), and the Bayesian confidence propagation neural network (BCPNN) to assess the significant association of Dexmedetomidine with AEs (Sakaeda et al., 2013). The ROR and PRR algorithms are frequentist (non-Bayesian) algorithms. Non-Bayesian algorithms generally exhibit high sensitivity but relatively low specificity. The possibility of false positives increases, especially when the number of AEs is low. The BCPNN algorithms are Bayesian algorithms, known for their stability and higher specificity, especially in situations with a small reporting volume, which could reduce the likelihood of false positives (Zou et al., 2023). In this study, the ROR algorithm, PRR algorithm, and BCPNN algorithm are combined to leverage the strengths of multiple algorithms and reduce the result bias that may arise from relying on a single algorithm. Before calculating these equations, the values of four variables (a, b, c, and d) need to be determined. Refer to a two-by-two contingency table (Table 1), where a represents the number of target AE to the target drug, b represents the number of other AEs to the target drug, c represents the number of target AE to other drugs, and d represents the number of other AEs to other drugs. The specific formulas for the three algorithms are provided below.

**TABLE 1:** A two-by-two contingency table.

	Taget AE	Other AEs	Total
Taget drug	a	b	a + b
Other drugs	c	d	c + d
Total	a + c	b + d	N = a + b + c + d

#### 2.2.1 ROR

$$ROR=\frac{\left(a/c\right)}{\left(b/d\right)}=\frac{ad}{bc}$$

$$SE\left(lnROR\right)=\sqrt{\left(\frac{1}{a}+\frac{1}{b}+\frac{1}{c}+\frac{1}{d}\right)}$$

$$95\%\text{CI}=\text{eln}\left(\text{ROR}\right)\pm 1.96\sqrt{\left(\frac{1}{a}+\frac{1}{b}+\frac{1}{c}+\frac{1}{d}\right)}$$

If N≥3 and the lower limit of 95%CI>1 , then generate a signal.

#### 2.2.2 PRR

$$\text{PRR}=\frac{a/\left(a+b\right)}{c/\left(c+d\right)}$$

$$X^{2}=\frac{\left(a+b+c+d\right){\left(ad-bc\right)}^{2}}{\left(a+b\right)\left(c+d\right)\left(a+c\right)\left(b+d\right)}$$

If PRR≥2 , $X^{2}\geq 4$ , N≥3 , then generate a signal.

#### 2.2.3 BCPNN

$$\text{IC}=\log_{2}\frac{a\left(a+b+c+d\right)}{\left(a+b\right)\left(a+c\right)}$$

$$95\%\text{CI}=E\left(\text{IC}\right)\pm 2\sqrt{V\left(\text{IC}\right)}$$

If IC025>0 (IC025: the lower limit of 95%CI), then generate a signal.

The time to event onset of Dexmedetomidine was calculated as the gap between EVENT_DT (date of onset of AEs) and START_DT (date of Dexmedetomidine use initiation). In addition, inaccurate dates, missing dates or input error reports were excluded. R software (version 4.3.2) and R Studio were utilized for data processing and statistical analysis.

## 3 Result

### 3.1 Basic population characteristics

Between the first quarter of 2004 and the third quarter of 2023, the FAERS database received a total of 20,214,432 reports. After deduplication, there were 1,913 cases in which Dexmedetomidine was the PS drug. Table 2 demonstrates the basic population characteristics with Dexmedetomidine-related AEs. The majority of patients were male (48.8%), with only 28.7% being female, while the gender of 22.4% of patients was unknown. The weight of the patients was predominantly distributed between 50 and 100 kg (17.1%). Regarding age, AEs were more prevalent among patients aged 18–6 years (31.2%) than in those over 65 years (19.2%). Most of the adverse event cases were submitted by Physician (30.8%), Health professional (25.3%), Other health-professional (24.2%), Pharmacist (14.4%) and only 2.3% of the cases were submitted by consumers.

**TABLE 2:** Characteristics of all cases associated with Dexmedetomidine from 2004 Q1-2023 Q3.

	Case Numbers	Percentage (%)
Overall	1910	
Gender		
Female	549	28.7
Male	933	48.8
Missing	428	22.4
Weight (kg)		
<50	204	10.7
>100	73	3.8
50–100	326	17.1
Missing	1,307	68.4
Age (year)		
≤17	300	15.7
≥86	19	1.0
18–64	596	31.2
65–85	367	19.2
Missing	628	32.9
Reporter		
Consumer	43	2.3
Health professional	484	25.3
Physician	589	30.8
Other health-professional	463	24.2
Pharmacist	275	14.4
Registered Nurse	1	0.1
Missing	55	2.9

### 3.2 Time to event onset

**FIGURE 2:**
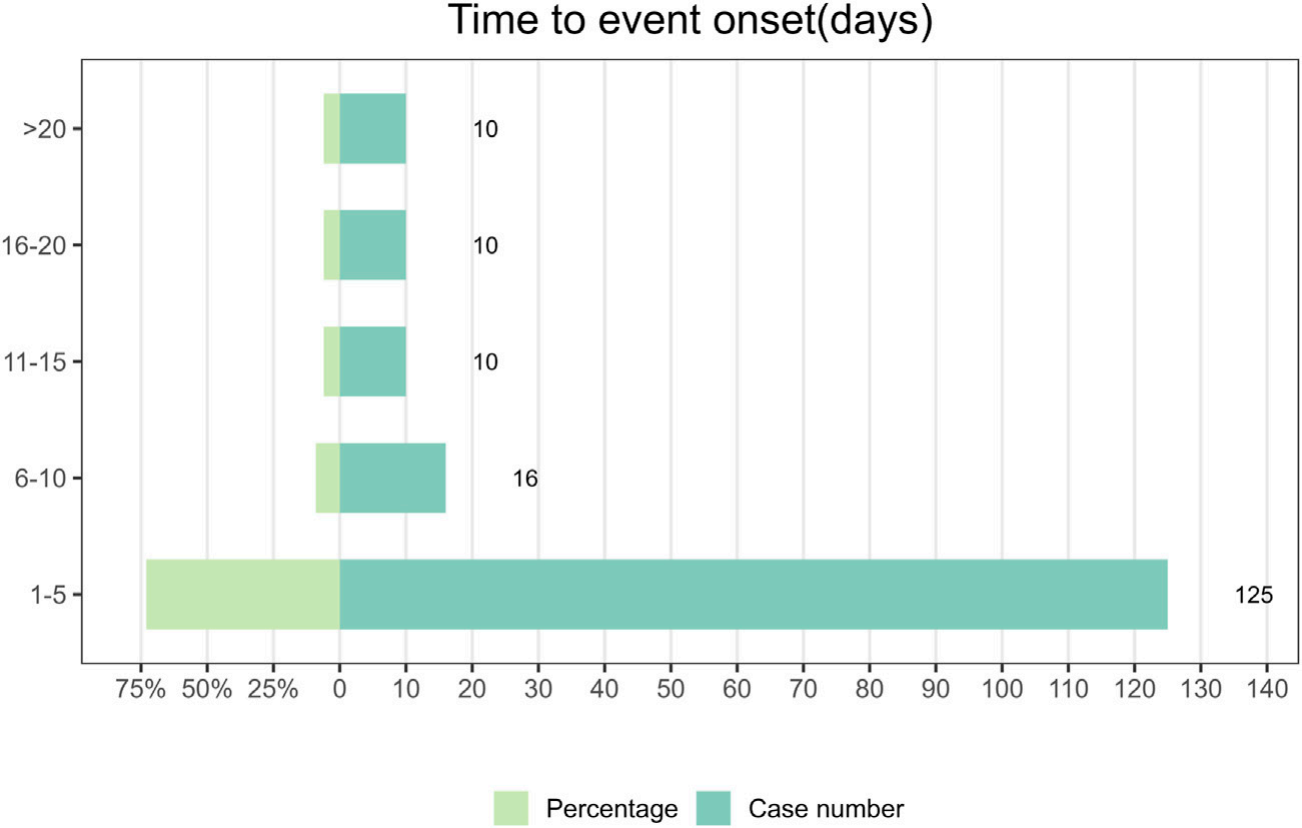
Time to event onset.

Between the first quarter of 2004 and the third quarter of 2023, a total of 176 reports documenting information on the timing of AEs were collected. The median time to the onset of AEs was 2 days, with an interquartile range of 1–6 days. In Figure 2, it was found that the majority of Dexmedetomidine AEs occurred within 5 days (n = 125, 73%) after drug administration. AEs rarely occurred within the time intervals of 6–10 days, 11–15 days, 16–20 days, and >20 days. Among these, there were 16 cases reported in the 6–10 days, accounting for 9% of the total, and 10 cases each in the remaining three categories, each representing 6% of the total. Because Dexmedetomidine is administered intravenously, concentrating its effects over a short period of time and dissipating quickly upon discontinuation. This means that adverse reactions usually occur shortly after administration. It also emphasizes the significance of early detection of AEs in patients shortly after drug administration.

### 3.3 Signal strength at the SOC level in FAERS database

**FIGURE 3:**
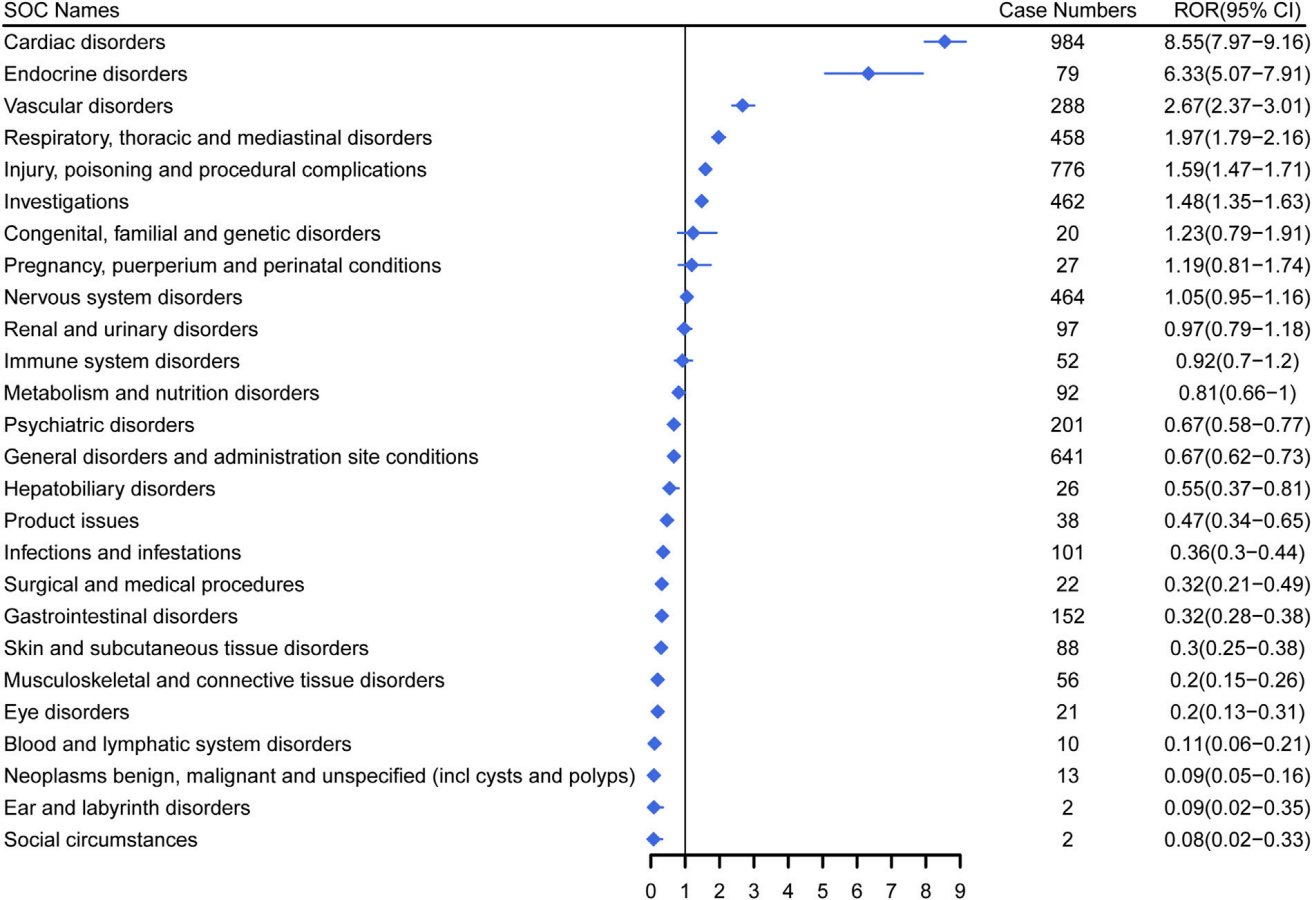
Signal strength of Dexmedetomidine at the system organ class (SOC) level in FAERS database. SOC, system organ class; ROR, reporting odds ratio.

Figure 3 demonstrates the signal strength of Dexmedetomidine at the SOC level in FAERS database. AEs related to Dexmedetomidine were observed in 26 organ systems. Among them, Dexmedetomidine was most frequently reported in cardiac disorders (n = 984) and exhibited the strongest signal [ROR (95%CI) = 8.55 (7.97–9.16)]. Several other SOCs have also shown strong signals, including Endocrine disorders [n = 79, ROR (95%CI) = 6.33 (5.07–7.91)], Vascular disorders [n = 288, ROR (95%CI) = 2.67 (2.37–3.01)], Respiratory, thoracic and mediastinal disorders [n = 458, ROR (95%CI) = 1.97 (1.79–2.16)], Injury, poisoning and procedural complications [n = 776, ROR (95%CI) = 1.59 (1.47–1.71)], Investigations [n = 462, ROR (95%CI) = 1.48 (1.35–1.63)], Congenital, familial and genetic disorders [n = 20, ROR (95%CI) = 1.23 (0.79–1.91)] and Pregnancy, puerperium and perinatal conditions [n = 27, ROR (95%CI) = 1.19 (0.81–1.74)]. However, Congenital, familial and genetic disorders and Pregnancy, puerperium and perinatal conditions did not have statistical significance due to the lower limit of ROR (95%CI) < 1.

### 3.4 Subgroup analysis

Because cardiac disorders were reported most frequently and with the highest signal intensity in the SOC, we chose cardiac disorders as the outcome variable for subgroup analyses and further analyzed the effects of age and sex on Dexmedetomidine AEs (Table 3). According to the results of univariate and multivariate logistic regression analyses, age is an influential factor in the occurrence of cardiac disorders. For those aged 18–64 years (OR = 1.09, *P* = 0.510), the risk of cardiac disorders was slightly increased relative to those under 18 years of age, but not statistically significant. However, for those over 65 years of age (OR = 1.69, *P* < 0.001), the risk of cardiac disorders was significantly higher than for those under 18 years of age. Regarding gender, for men (OR = 1.22, *P* = 0.068), there was a slight increase in the risk of cardiac disorders relative to women, approaching a level of statistical significance that may be clinically significant but has not yet reached significance.

**TABLE 3:** Influence of demographic variables on cardiac disorders in logistic regression analysis.

Cardiac disorders		Not occurred	Occurred	OR (univariable)	OR (multivariable)
Age	<18 years	188 (57.7)	138 (42.3)	—	—
	18–64 years	390 (55.5)	313 (44.5)	1.09 (0.84–1.43, *p* = 0.510)	1.08 (0.83–1.42, *p* = 0.550)
	>65 years	210 (44.7)	260 (55.3)	1.69 (1.27–2.25, *p* < 0.001)	1.68 (1.26–2.23, *p* < 0.001)
Sex	Female	288 (55.8)	228 (44.2)	—	—
	Male	500 (50.9)	483 (49.1)	1.22 (0.99–1.51, *p* = 0.068)	1.21 (0.98–1.51, *p* = 0.078)

### 3.5 Signal strength associated with Dexmedetomidine at the prefer term level

**FIGURE 4:**
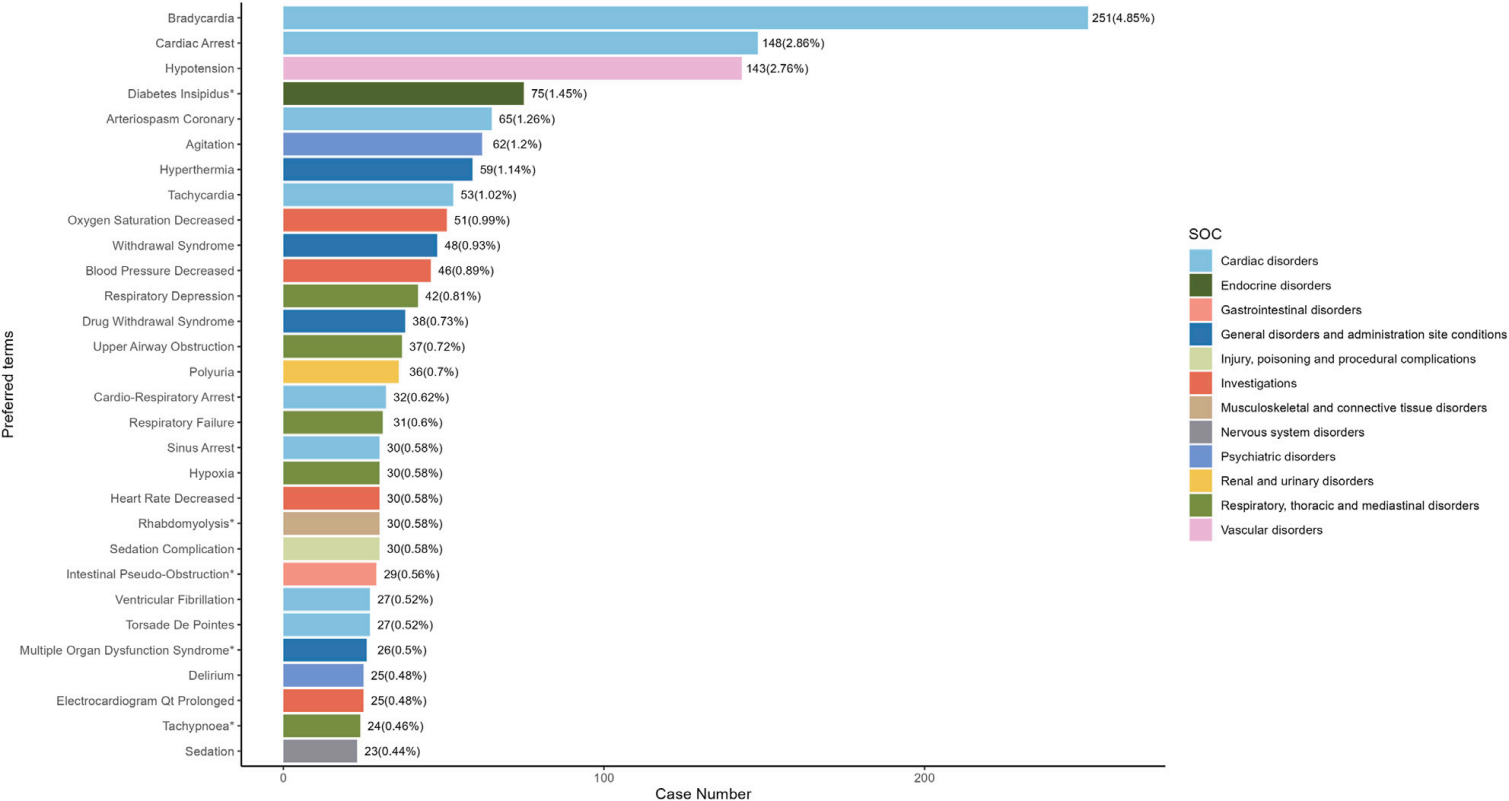
The top 30 adverse events of Dexmedetomidine at the preferred terms level in FAERS database. PT, preferred term of the Medical Dictionary for Regulatory Activities; ROR, reporting odds ratio; *indicates new signals of Dexmedetomidine adverse events from FAERS Database.

**FIGURE 5:**
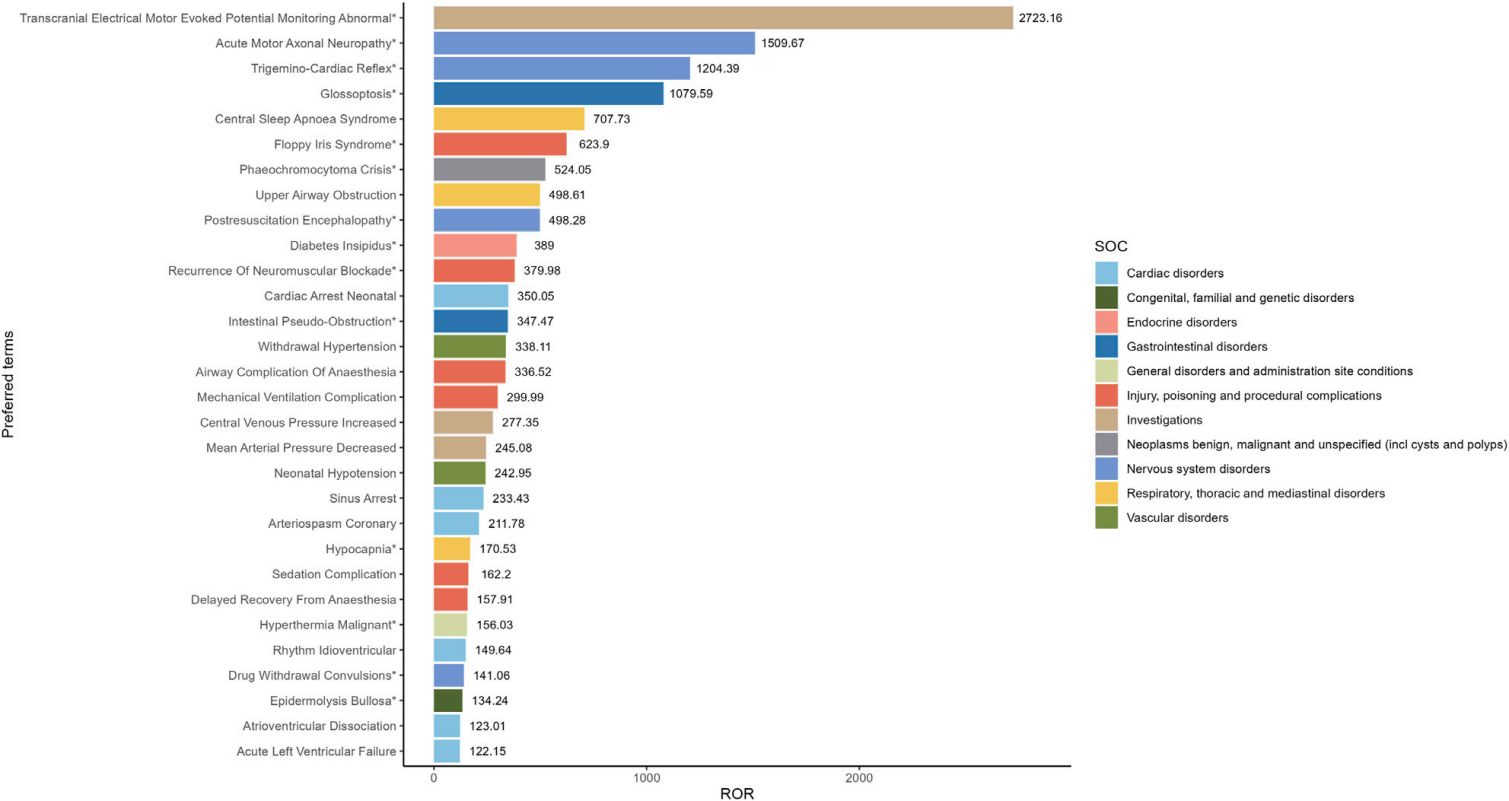
The top 30 signal strength of AEs of Dexmedetomidine ranked by ROR at the preferred terms level in the FAERS database. PT, preferred term of the Medical Dictionary for Regulatory Activities; ROR, reporting odds ratio; *indicates new signals of Dexmedetomidine adverse events from FAERS Database.

Our study employed three algorithms to analyze drug responses and assess whether they met various screening criteria. A total of 201 positive signals were detected (Supplementary Table S1). The signals not related to the AEs of the drug itself, such as product problems, social environment, off-label use, drug interaction, drug ineffective and overdose were excluded. The signals were ranked by case reports (Figure 4) and ROR (Figure 5) respectively, and the PTs within the top 30 rankings were selected for presentation. PTs seen to occur with higher frequency include Bradycardia, Cardiac Arrest, Hypotension, Diabetes Insipidus, Arteriospasm Coronary, Agitation, Hyperthermia, Tachycardia, Oxygen Saturation Decreased and Withdrawal Syndrome (Figure 4). As shown in Figure 5, some PTs with high ROR signals were observed, such as Transcranial Electrical Motor Evoked Potential Monitoring Abnormal, Acute Motor, Axonal Neuropathy, Trigemino-Cardiac Reflex, Glossoptosis, Central Sleep, Apnoea Syndrome, Floppy Iris Syndrome, Phaeochromocytoma Crisis, Upper Airway Obstruction, Postresuscitation Encephalopathy and Diabetes Insipidus.

### 3.6 New AEs associated with Dexmedetomidine

**FIGURE 6:**
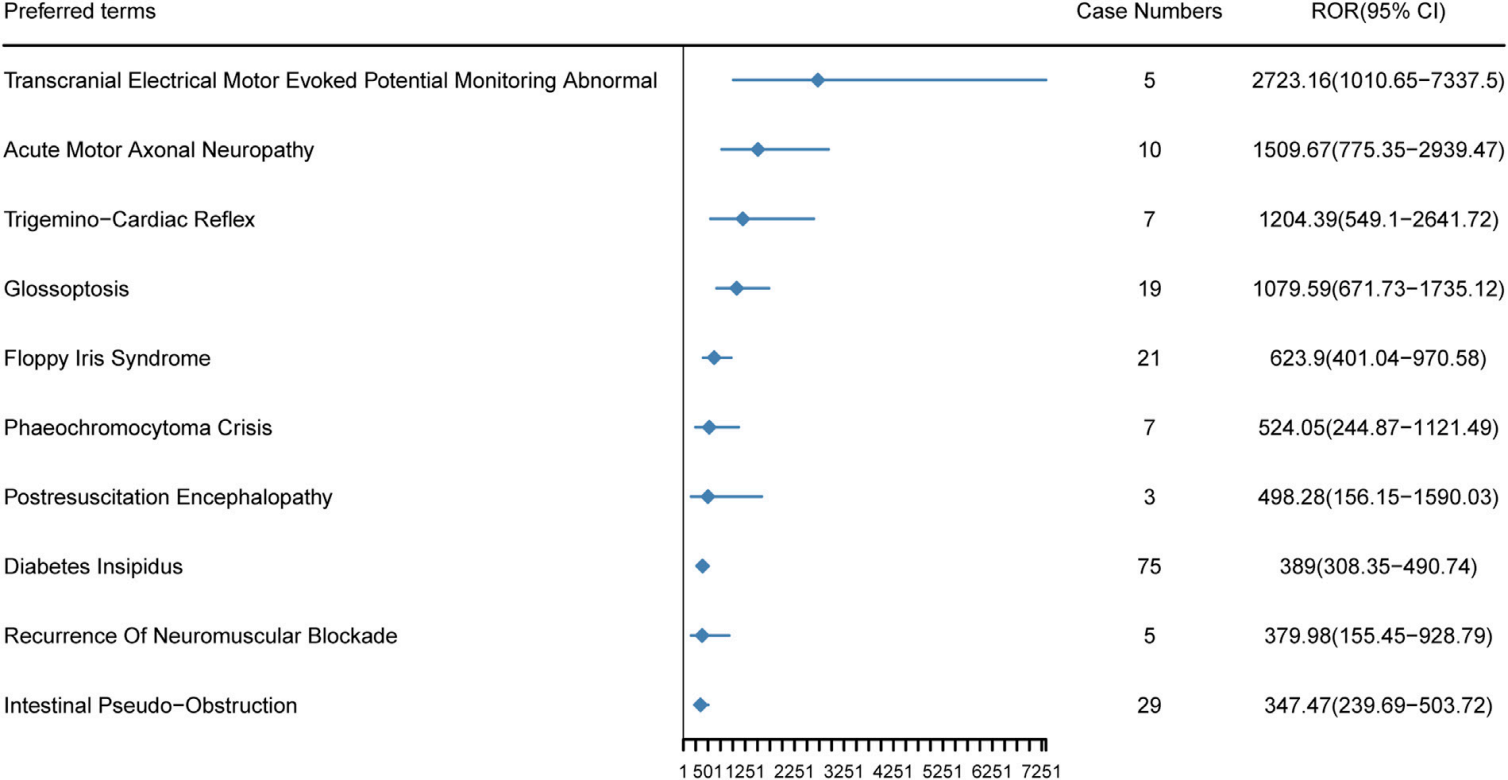
The top 10 new AEs of Dexmedetomidine ranked by ROR at the preferred terms level in the FAERS database. PT, preferred term of the Medical Dictionary for Regulatory Activities; ROR, reporting odds ratio.

In our study, 57 new AEs of Dexmedetomidine not mentioned in the drug insert were identified, and these new signs were ranked by ROR (Supplementary Table S2). Among these, the top 10 new AEs are illustrated in Figure 6, including Transcranial Electrical Motor Evoked Potential Monitoring Abnormal [ROR (95%CI) = 2,723.16 (1,010.65–7,337.50)], Acute Motor Axonal Neuropathy [ROR (95%CI) = 1,509.67 (775.35–2,939.47)], Trigemino-Cardiac Reflex [ROR (95%CI) = 1,204.39 (549.10–2,641.72)], Glossoptosis [ROR (95%CI) = 1,079.59 (671.73–1735.12)], Floppy Iris Syndrome [ROR (95%CI) = 623.90 (401.04–970.58)], Phaeochromocytoma Crisis [ROR (95%CI) = 524.05 (244.87–1,121.49)], Postresuscitation Encephalopathy [ROR (95%CI) = 498.28 (156.15–1,590.03)], Diabetes Insipidus [ROR (95%CI) = 389.00 (308.35–490.74)], Recurrence Of Neuromuscular Blockade [ROR (95%CI) =379.98 (155.45–928.79)] and Intestinal Pseudo-Obstruction [ROR (95%CI =347.47 (239.69–503.72)]. The new AEs data provides us with more comprehensive information, aiding in a deeper understanding of the safety profile and potential risks associated with Dexmedetomidine. By analyzing these data, we can assess a wide range of reactions that the drug may exhibit in real-world applications, offering crucial insights for clinicians and researchers.

## 4 Discussion

Dexmedetomidine acts as a highly selective agonist for $\alpha_{2}$ adrenergic receptor. It is extensively employed in clinical settings for sedation and anesthesia owing to its minimal impact on the respiratory system, effective stress suppression, and maintenance of hemodynamic stability, with its usage increasing year by year. Therefore, it is crucial to monitor the safety profile of Dexmedetomidine. This study leverages the publicly available and largest pharmacovigilance database, the FAERS database, to offer real-world data on the safety of Dexmedetomidine, guiding its rational clinical use. In this study, it was found that AEs of Dexmedetomidine occurred more often in male patients by analyzing 1913 reports of AEs. And based on the results of the subgroup analysis, we found that the risk of cardiac disorders was slightly higher in men than in women but was not an independent risk factor for cardiac disorders with Dexmedetomidine. This may be due to the fact that there are more males than females admitted to the ICU with a medical diagnosis or a non-obstetric surgical diagnosis. In addition, significantly more critically ill males than females received invasive treatments requiring sedation such as mechanical ventilation and pulmonary artery catheterization (Fowler et al., 2007; Modra et al., 2022). However, whether there are gender differences in the occurrence of Dexmedetomidine-associated AEs is controversial, and no mechanistic studies have been identified. Meanwhile, the results of our study showed that the risk of cardiac disorders was significantly higher in older people over the age of 65 than in younger people under the age of 18, suggesting that age is an important risk factor for cardiac disorders induced by Dexmedetomidine. Common cardiac disorders include bradycardia and hypotension, the occurrence of which is related to the drug dose (Ko et al., 2015). Therefore, when Dexmedetomidine is used in the elderly, it should be titrated at a low dose, and special attention needs to be paid to monitoring blood pressure and heart rate to minimize the incidence of adverse cardiovascular events. Furthermore, the majority of AEs in our study were reported by physicians, pharmacists, or other medical industry practitioners, which adds to the credibility of the AEs and corresponds to clinical reality. This may be because Dexmedetomidine is primarily used in ICU and for anesthesia sedation, and thus, it is mostly administered under the supervision of healthcare professionals. Analysis of the time to onset of AEs showed a median occurrence time of 2 days for Dexmedetomidine-associated AEs, with the majority occurring within 5 days of Dexmedetomidine use. This may be due to the fact that the time of onset of AEs is reported only in days rather than hours, leading to results that are not precise. In fact, Dexmedetomidine has a short distribution half-life and rapidly distributes throughout the body. The distribution half-life is approximately 6 min, and the elimination half-life ranges from 2.1 to 3.1 h in healthy populations. However, the elimination half-life is extended to 2.2–3.7 h in ICU patients (Weerink et al., 2017). Therefore, it is crucial to closely observe the vital signs of patients following the administration of the drug.

By analyzing the data, we found that the most common SOCs for Dexmedetomidine was cardiac disorders, which also had the strongest signal strength. And the next most common SOCs was vascular disorders. The most common AEs for cardiac disorders and vascular disorders are bradycardia and hypotension, respectively. The reason for this is that Dexmedetomidine, at high plasma concentrations, activates receptors in $\alpha_{2}$ -vascular smooth muscle, causing peripheral vasoconstriction thereby inducing hypertension, as well as reflexively lowering heart rate. Conversely, Dexmedetomidine activates receptors in $\alpha_{2}$ -vascular endothelial cells causing vasodilation at low plasma concentrations. It also agitates presynaptic $\alpha_{2}$ -adrenergic receptors, inhibits catecholamine release and increases vagal activity (Weerink et al., 2017). In a randomized trial and meta-analysis of Dexmedetomidine use in adult mechanical ventilation, 36 out of 77 trials reported bradycardia, while 40 reported hypotension following administration (Lewis et al., 2022). Furthermore, 71% of noncardiac critically ill patients experienced hemodynamic instability within 24 h of Dexmedetomidine sedation (Li et al., 2015). Previous studies have indicated that bradycardia and hypotension are more likely to occur during the rapid titration and loading infusion of Dexmedetomidine. Therefore, the occurrence of associated AEs can be reduced by increasing the titration interval and decreasing the loading infusion (Gerlach et al., 2009; Venn et al., 2003).

Based on the data analysis, except from cardiac and vascular disorders, we also observed a significant correlation between Dexmedetomidine and respiratory, thoracic, and mediastinal disorders. Common AEs associated with respiratory, thoracic, and mediastinal disorders were respiratory depression, upper airway obstruction, respiratory failure, and hypoxia. While previous studies have suggested that Dexmedetomidine has minimal respiratory depression, our real-world data indicate that respiratory-related AEs associated with Dexmedetomidine are still relatively common. This observation may be attributed to the role of $\alpha_{2}$ -adrenergic receptors in controlling ventilation (Hsu et al., 2004). In a study of Dexmedetomidine sedation given to healthy male volunteers, it was found that 9 out of 10 subjects experienced snoring (Gerlach et al., 2009). Therefore, careful monitoring of respiratory function at regular intervals is essential in the clinical application of Dexmedetomidine.

Our study also found that the most common AEs at the PT level included bradycardia, cardiac arrest, hypotension, diabetes insipidus, arteriospasm coronary, agitation. AEs to be aware of include arteriospasm coronary, and most studies have now concluded that the application of Dexmedetomidine during cardiac surgery improves the prognosis of patients (Ji et al., 2013). However, studies have also revealed that Dexmedetomidine may result in a reduction in coronary artery diameter (Kundra et al., 2016). In addition, Sato et al. (2022) published a case report describing transient coronary artery spasm in a patient who received postoperative sedation with Dexmedetomidine. This phenomenon is thought to be related to the ability of Dexmedetomidine to activate $\alpha_{2}$ - adrenergic receptors, causing coronary artery constriction and reducing coronary blood flow (Kundra et al., 2016). Therefore, Dexmedetomidine should be handled cautiously in patients with underlying cardiovascular disease.

Based on our study data, we also observed AEs of drug withdrawal syndrome associated with Dexmedetomidine. The drug withdrawal syndrome of Dexmedetomidine is characterized by tension, agitation, headache, elevated blood pressure, and increased catecholamine concentrations following abrupt discontinuation of an infusion of Dexmedetomidine (Infusion time ≥ 24 h). Kukoyi et al. (2013) documented two cases of adults with tachycardia, hypertension, agitation and anxiety following abrupt discontinuation of Dexmedetomidine after a prolonged infusion. Their signs and symptoms improved after receiving oral clonidine (Kukoyi et al., 2013). Risk factors for the drug withdrawal syndrome include duration of Dexmedetomidine infusion and cumulative volume. Notably, Dexmedetomidine exposure variables were not significantly correlated with withdrawal syndrome. Therefore, during clinical application of Dexmedetomidine, attention should be given to its infusion duration to prevent excessive use. Additionally, clonidine can be considered to alleviate the withdrawal symptoms of Dexmedetomidine (Knapp et al., 2024).

In our study, several new signals were identified. Particularly, Transcranial Electrical Motor Evoked Potential Monitoring Abnormal exhibited the highest signal intensity. The underlying mechanism by which Dexmedetomidine induces this phenomenon may be similar to that of clonidine. It may act by suppressing electrical responses in specific spinal neurons, inhibiting the activity of adrenergic neurons in the locus coeruleus, and decreasing activity in serotonergic neurons in the dorsal raphe nucleus (Wolff et al., 2007; Calderón et al., 2018). In addition, Mahmoud et al. (2010) also found that Dexmedetomidine significantly attenuated the amplitude of transcranial electrical motor evoked potential at plasma concentrations range from 0.6 to 0.8 ng/mL during spinal surgery. Our study adds to the evidence on real-world use, but a more in-depth exploration of the specific mechanisms requires further investigation through additional studies.

Secondly, diabetes insipidus as the most common PT in new signals, Van Decar et al. (2022) found that Dexmedetomidine was significantly associated with perioperative diabetes insipidus. In dog and rat studies, it was shown that the mechanism is related to the reduction of central arginine vasopressin (AVP) release by Dexmedetomidine, as well as the reduction of renal response to AVP (Van Decar et al., 2022). However, the specific mechanism in humans is not clear, and further studies on Dexmedetomidine-induced diabetes insipidus in humans are still needed to elucidate the underlying mechanisms in the future.

An interesting finding is that we observed a significant association between Floppy Iris Syndrome and Dexmedetomidine. As of now, there are no reported cases in the literature of Dexmedetomidine causing Floppy Iris Syndrome. The pathogenesis of this condition remains incompletely understood at present, and it may be associated with the impact on the sympathetic nervous system by $\alpha_{2}$ - adrenergic receptor agonists, leading to pupil dilation (Wolfran et al., 2022). This PT is not listed in the drug insert, so the safety of Dexmedetomidine in patients with ocular disease is unclear. However, our findings suggest that in practical clinical applications, greater attention is needed when using Dexmedetomidine in patients with ocular diseases.

Overall, based on our findings, Dexmedetomidine can cause bradycardia and hypotension and should therefore be avoided in patients with severe heart block or bradycardia. As well as in patients with severe hypotension or poorly controlled blood pressure, Dexmedetomidine should be used with caution to avoid exacerbating symptoms of hypotension. Although Dexmedetomidine is less inhibitory to the respiratory system, AEs associated with respiratory depression, upper airway obstruction, and hypoxemia have been observed. Therefore, respiratory function should be monitored when Dexmedetomidine is administered, especially in patients with respiratory disease. In addition, our study suggests that Dexmedetomidine may cause diabetes insipidus by a mechanism that may involve an effect on antidiuretic hormone release. Therefore, the water-electrolyte balance should be monitored when Dexmedetomidine is administered. When using Dexmedetomidine in clinical practice, we recommend that the dose be adjusted according to the patient’s age, condition, and drug interactions. In particular, lower doses should be used and gradually adjusted for elderly patients. And continuous monitoring of heart rate, blood pressure, and respiratory function is required during use for the timely detection and management of AEs. In addition, after prolonged use, the medication should be discontinued by gradually reducing the dose to minimize or avoid withdrawal syndrome. If withdrawal syndrome occurs, drugs such as clonidine may be considered to alleviate withdrawal symptoms.

Certainly, this study has several limitations. Firstly, the FAERS database is a spontaneous reporting system with a diverse range of reporting sources. Therefore, there may be omissions, delayed reporting, missing information and misreporting, which could impact the analysis of results. Secondly, we were unable to calculate the incidence of AEs due to the unavailability of the overall number of people on medication. Thirdly, the three calculations we employed (ROR, PRR, and BCPNN) can only reflect the statistical correlation between drugs and AEs, but cannot determine the causal relationship between drugs and AEs. Therefore, more research experiments are still needed to establish the relationship between them. In spite of these limitations, the FAERS database still provides a substantial volume of data samples and relevant information on drugs in real-world applications. This helps researchers and clinicians identify potential safety issues following the release of a medication on the market, thereby reducing the risk associated with clinical drug use.

## 5 Conclusion

In summary, this study analyzed the real-world safety of Dexmedetomidine through the FAERS database. The common AEs include bradycardia, cardiac arrest, hypotension, diabetes insipidus, arteriospasm coronary and agitation. In addition, this study identified 57 new signals not mentioned in the drug insert. Among them, transcranial electrical motor evoked potential monitoring abnormal, acute motor axonal neuropathy, trigemino-cardiac reflex, glossoptosis, floppy iris syndrome, phaeochromocytoma crisis, postresuscitation encephalopathy, and diabetes insipidus showed a significant correlation with Dexmedetomidine. Therefore, physicians should closely monitor these common AEs and new AEs with high signal intensity during clinical use.

## Data Availability

The original contributions presented in the study are included in the article/Supplementary Material, further inquiries can be directed to the corresponding authors.
