# Supplementary material for: Dexmedetomidine: a real-world safety analysis based on FDA adverse event reporting system database
Source: Front Pharmacol. 2024 Aug 23;15:1419196. doi: 10.3389/fphar.2024.1419196 (PMC11377849; doi:10.3389/fphar.2024.1419196)
Supplement: Supplementary file 1 [file Table1.DOCX]

**Supplementary Table 1. Signal strength of AEs of Dexmedetomidine at the preferred terms level in FAERS database.**

| **PT** | **Case Reports** | **ROR(95%CI)** | **PRR(X^2^)** | **IC(IC025)** |
| --- | --- | --- | --- | --- |
| Bradycardia | 251 | 56.66(49.90-64.35) | 53.96(12987.25) | 5.75(4.08) |
| Cardiac Arrest | 148 | 21.08(17.90-24.83) | 20.51(2744.40) | 4.36(2.69) |
| Hypotension | 143 | 8.58(7.27-10.14) | 8.37(930.74) | 3.06(1.40) |
| Product Administered To Patient Of Inappropriate Age | 95 | 86.82(70.81-106.45) | 85.24(7842.61) | 6.40(4.73) |
| Diabetes Insipidus | 75 | 389.00(308.35-490.74) | 383.37(27526.81) | 8.53(6.86) |
| Product Use Issue | 69 | 4.66(3.67-5.91) | 4.61(195.52) | 2.20(0.54) |
| Drug Interaction | 67 | 4.97(3.91-6.33) | 4.92(209.74) | 2.30(0.63) |
| Arteriospasm Coronary | 65 | 211.78(165.40-271.18) | 209.13(13183.35) | 7.68(6.01) |
| Agitation | 62 | 9.57(7.45-12.30) | 9.47(469.76) | 3.24(1.58) |
| Hyperthermia | 59 | 91.88(71.00-118.90) | 90.84(5194.76) | 6.49(4.83) |
| Tachycardia | 53 | 7.10(5.42-9.31) | 7.04(274.82) | 2.81(1.15) |
| Oxygen Saturation Decreased | 51 | 11.69(8.87-15.40) | 11.58(492.89) | 3.53(1.87) |
| Withdrawal Syndrome | 48 | 14.48(10.89-19.24) | 14.35(595.66) | 3.84(2.17) |
| Blood Pressure Decreased | 46 | 8.23(6.15-11.00) | 8.16(289.26) | 3.03(1.36) |
| Respiratory Depression | 42 | 39.86(29.41-54.04) | 39.55(1572.08) | 5.30(3.63) |
| Drug Withdrawal Syndrome | 38 | 4.45(3.23-6.12) | 4.42(100.72) | 2.14(0.48) |
| Upper Airway Obstruction | 37 | 498.61(357.96-694.52) | 495.05(17365.35) | 8.88(7.21) |
| Polyuria | 36 | 51.89(37.35-72.08) | 51.53(1774.75) | 5.68(4.01) |
| Cardio-Respiratory Arrest | 32 | 8.64(6.10-12.24) | 8.60(214.73) | 3.10(1.44) |
| Respiratory Failure | 31 | 4.95(3.48-7.04) | 4.92(96.99) | 2.30(0.63) |
| Sinus Arrest | 30 | 233.43(162.36-335.62) | 232.08(6743.02) | 7.82(6.16) |
| Hypoxia | 30 | 10.40(7.27-14.90) | 10.35(253.29) | 3.37(1.70) |
| Hear Rate Decreased | 30 | 9.83(6.87-14.08) | 9.78(236.37) | 3.29(1.62) |
| Rhabdomyolysis | 30 | 8.54(5.96-12.23) | 8.50(198.40) | 3.09(1.42) |
| Sedation Complication | 30 | 162.20(112.96-232.90) | 161.26(4700.79) | 7.31(5.64) |
| Intestinal Pseudo-Obstruction | 29 | 347.47(239.69-503.72) | 345.53(9623.04) | 8.38(6.71) |
| Ventricular Fibrillation | 27 | 27.87(19.09-40.71) | 27.73(693.94) | 4.79(3.12) |
| Torsade De Pointes | 27 | 40.17(27.50-58.68) | 39.96(1021.64) | 5.31(3.65) |
| Multiple Organ Dysfunction Syndrome | 26 | 6.87(4.67-10.10) | 6.84(129.61) | 2.77(1.11) |
| Delirium | 25 | 8.90(6.01-13.19) | 8.86(174.36) | 3.15(1.48) |
| Electrocardiogram Qt Prolonged | 25 | 8.21(5.54-12.16) | 8.17(157.36) | 3.03(1.36) |
| Tachypnoea | 24 | 21.80(14.59-32.57) | 21.70(473.01) | 4.44(2.77) |
| Sedation | 23 | 11.25(7.47-16.96) | 11.21(213.69) | 3.49(1.82) |
| Anaphylactic Shock | 23 | 11.26(7.47-16.96) | 11.21(213.72) | 3.49(1.82) |
| Drug Withdrawal Convulsions | 22 | 141.06(92.52-215.06) | 140.46(3003.30) | 7.11(5.45) |
| Obstructive Airways Disorder | 21 | 22.48(14.64-34.53) | 22.39(428.32) | 4.48(2.82) |
| Floppy Iris Syndrome | 21 | 623.90(401.04-970.58) | 621.37(12230.84) | 9.19(7.52) |
| Sinus Bradycardia | 20 | 23.85(15.36-37.01) | 23.76(435.00) | 4.57(2.90) |
| Respiratory Arrest | 20 | 7.85(5.06-12.19) | 7.83(119.06) | 2.97(1.30) |
| Atrioventricular Block Complete | 20 | 34.95(22.51-54.27) | 34.82(654.78) | 5.12(3.45) |
| Hypernatraemia | 20 | 48.39(31.16-75.16) | 48.21(920.16) | 5.58(3.92) |
| Glossoptosis | 19 | 1079.59(671.73-1735.12) | 1075.63(18380.44) | 9.92(8.24) |
| Hyperthermia Malignant | 19 | 156.03(99.09-245.69) | 155.46(2870.35) | 7.26(5.59) |
| Laryngospasm | 19 | 80.57(51.25-126.65) | 80.28(1475.49) | 6.32(4.65) |
| Pneumonia Aspiration | 18 | 8.65(5.44-13.74) | 8.62(121.22) | 3.11(1.44) |
| Atrioventricular Block Second Degree | 17 | 64.10(39.75-103.35) | 63.89(1045.62) | 5.99(4.32) |
| Restlessness | 17 | 5.35(3.32-8.62) | 5.34(59.94) | 2.42(0.75) |
| Ventricular Tachycardia | 17 | 11.92(7.40-19.20) | 11.89(169.33) | 3.57(1.90) |
| Unresponsive To Stimuli | 15 | 6.82(4.11-11.32) | 6.80(74.22) | 2.77(1.10) |
| Circulatory Collapse | 15 | 9.89(5.95-16.41) | 9.86(119.34) | 3.30(1.63) |
| Hyperpyrexia | 15 | 51.01(30.69-84.79) | 50.87(729.58) | 5.66(3.99) |
| Neuroleptic Malignant Syndrome | 14 | 13.80(8.16-23.32) | 13.76(165.51) | 3.78(2.11) |
| Cardiogenic Shock | 14 | 12.53(7.41-21.18) | 12.50(147.98) | 3.64(1.98) |
| Haemodynamic Instability | 14 | 23.57(13.94-39.85) | 23.51(301.06) | 4.55(2.89) |
| Pulmonary Oedema | 13 | 3.37(1.95-5.81) | 3.36(21.60) | 1.75(0.08) |
| Depressed Level Of Consciousness | 13 | 3.79(2.20-6.54) | 3.79(26.67) | 1.92(0.25) |
| Generalised Tonic-Clonic Seizure | 13 | 5.86(3.40-10.10) | 5.85(52.21) | 2.55(0.88) |
| Shock | 13 | 6.97(4.04-12.01) | 6.95(66.25) | 2.80(1.13) |
| Pulseless Electrical Activity | 12 | 28.39(16.10-50.06) | 28.33(315.46) | 4.82(3.15) |
| Ventricular Extrasystoles | 12 | 12.96(7.35-22.84) | 12.93(131.91) | 3.69(2.02) |
| Myocardial Ischaemia | 12 | 11.12(6.31-19.60) | 11.10(110.14) | 3.47(1.80) |
| Accident At Work | 12 | 63.95(36.23-112.89) | 63.80(737.06) | 5.99(4.32) |
| Skin Laceration | 12 | 6.74(3.82-11.87) | 6.72(58.45) | 2.75(1.08) |
| Delayed Recovery From Anaesthesia | 12 | 157.91(89.21-279.50) | 157.54(1837.07) | 7.28(5.61) |
| Dyskinesia | 12 | 3.42(1.94-6.03) | 3.42(20.53) | 1.77(0.11) |
| Aspiration | 11 | 12.43(6.88-22.47) | 12.41(115.26) | 3.63(1.96) |
| Cerebral Infarction | 11 | 5.14(2.84-9.29) | 5.13(36.58) | 2.36(0.69) |
| Product Closure Removal Difficult | 11 | 164.83(90.77-299.29) | 164.48(1757.83) | 7.34(5.67) |
| Hypertensive Crisis | 11 | 11.36(6.28-20.53) | 11.34(103.59) | 3.50(1.83) |
| Myoclonus | 11 | 10.82(5.99-19.56) | 10.80(97.73) | 3.43(1.76) |
| Apnoea | 11 | 15.84(8.76-28.63) | 15.80(152.31) | 3.98(2.31) |
| Restless Legs Syndrome | 11 | 6.97(3.86-12.60) | 6.96(56.13) | 2.80(1.13) |
| Posterior Reversible Encephalopathy Syndrome | 10 | 12.10(6.51-22.52) | 12.08(101.53) | 3.59(1.93) |
| Accidental Overdose | 10 | 3.40(1.83-6.33) | 3.40(16.94) | 1.77(0.10) |
| Stress Cardiomyopathy | 10 | 22.82(12.26-42.48) | 22.78(207.79) | 4.51(2.84) |
| Nodal Rhythm | 10 | 75.14(40.31-140.06) | 74.99(724.52) | 6.22(4.55) |
| Acute Respiratory Distress Syndrome | 10 | 6.66(3.58-12.39) | 6.65(48.01) | 2.73(1.07) |
| Acute Motor Axonal Neuropathy | 10 | 1509.67(775.35-2939.47) | 1506.76(13041.24) | 10.35(8.65) |
| Blood Creatine Phospho kinase Increased | 9 | 3.40(1.77-6.53) | 3.39(15.18) | 1.76(0.10) |
| Respiratory Acidosis | 9 | 28.10(14.60-54.09) | 28.05(234.15) | 4.81(3.14) |
| Atrioventricular Block | 9 | 13.63(7.08-26.22) | 13.60(104.97) | 3.76(2.10) |
| Cardiac Tamponade | 9 | 22.26(11.57-42.83) | 22.22(181.98) | 4.47(2.80) |
| Electrocardiogram St Segment Elevation | 9 | 30.36(15.77-58.45) | 30.31(254.33) | 4.92(3.25) |
| Respiratory Distress | 9 | 3.78(1.97-7.28) | 3.78(18.39) | 1.92(0.25) |
| Bradyarrhythmia | 9 | 63.80(33.11-122.95) | 63.69(551.77) | 5.98(4.32) |
| Hypocalcaemia | 9 | 5.74(2.99-11.04) | 5.73(35.16) | 2.52(0.85) |
| Premature Baby | 9 | 3.42(1.78-6.58) | 3.41(15.37) | 1.77(0.10) |
| Bradycardia Neonatal | 9 | 81.83(42.44-157.79) | 81.69(711.42) | 6.34(4.67) |
| Atelectasis | 9 | 10.40(5.41-20.01) | 10.39(76.28) | 3.38(1.71) |
| Post Procedural Complication | 9 | 5.37(2.79-10.32) | 5.36(31.91) | 2.42(0.75) |
| Blood Albumin Decreased | 9 | 13.01(6.76-25.03) | 12.99(99.49) | 3.70(2.03) |
| Electrolyte Imbalance | 8 | 8.57(4.28-17.16) | 8.56(53.38) | 3.10(1.43) |
| Supraventricular Tachycardia | 8 | 9.57(4.78-19.16) | 9.56(61.26) | 3.26(1.59) |
| Procedural Complication | 8 | 11.36(5.67-22.73) | 11.34(75.35) | 3.50(1.84) |
| Hypopnoea | 8 | 28.69(14.33-57.46) | 28.65(212.85) | 4.84(3.17) |
| Neonatal Hypotension | 8 | 242.95(120.40-490.23) | 242.57(1878.12) | 7.89(6.21) |
| Hiccups | 8 | 11.97(5.98-23.96) | 11.95(80.21) | 3.58(1.91) |
| Wrong Product Administered | 8 | 5.81(2.91-11.64) | 5.81(31.82) | 2.54(0.87) |
| Prinzmetal Angina | 8 | 99.71(49.66-200.19) | 99.56(772.69) | 6.62(4.95) |
| Hypothermia | 8 | 9.33(4.66-18.67) | 9.32(59.33) | 3.22(1.55) |
| Respiratory Disorder | 8 | 3.18(1.59-6.36) | 3.18(11.92) | 1.67(0.00) |
| Encephalopathy | 7 | 3.43(1.63-7.19) | 3.42(12.01) | 1.78(0.11) |
| Ileus | 7 | 6.94(3.30-14.56) | 6.93(35.49) | 2.79(1.13) |
| Airway Complication Of Anaesthesia | 7 | 336.52(158.34-715.20) | 336.07(2260.91) | 8.34(6.67) |
| Infusion Site Extravasation | 7 | 12.68(6.04-26.63) | 12.67(75.14) | 3.66(1.99) |
| Extravasation | 7 | 19.14(9.11-40.20) | 19.12(119.97) | 4.25(2.59) |
| Incorrect Drug Administration Rate | 7 | 22.12(10.53-46.47) | 22.09(140.67) | 4.46(2.80) |
| Seizure Like Phenomena | 7 | 54.87(26.09-115.40) | 54.80(367.69) | 5.77(4.10) |
| Acute Left Ventricular Failure | 7 | 122.15(57.94-257.54) | 121.99(829.65) | 6.91(5.24) |
| Trigemino-Cardiac Reflex | 7 | 1204.39(549.10-2641.72) | 1202.76(7486.01) | 10.07(8.36) |
| Central Sleep Apnoea Syndrome | 7 | 707.73(328.49-1524.81) | 706.78(4601.40) | 9.36(7.67) |
| Phaeochromocytoma Crisis | 7 | 524.05(244.87-1121.49) | 523.34(3464.28) | 8.96(7.27) |
| Prothrombin Time Prolonged | 7 | 12.14(5.78-25.49) | 12.12(71.36) | 3.60(1.93) |
| Withdrawal Hypertension | 6 | 338.11(149.77-763.32) | 337.72(1947.21) | 8.35(6.67) |
| Product Package Associated Injury | 6 | 34.54(15.49-77.04) | 34.51(194.53) | 5.10(3.44) |
| Product Packaging Confusion | 6 | 29.55(13.25-65.88) | 29.51(164.80) | 4.88(3.21) |
| Haematocrit Decreased | 6 | 3.30(1.48-7.35) | 3.30(9.60) | 1.72(0.05) |
| Premature Delivery | 6 | 4.34(1.95-9.67) | 4.34(15.39) | 2.12(0.45) |
| Product Label Confusion | 6 | 13.45(6.04-29.97) | 13.44(68.98) | 3.75(2.08) |
| Hypocapnia | 6 | 170.53(76.05-382.39) | 170.33(992.75) | 7.39(5.71) |
| Hypoalbuminaemia | 6 | 9.61(4.31-21.40) | 9.60(46.17) | 3.26(1.59) |
| Hypoxic-Ischaemic Encephalopathy | 5 | 14.97(6.22-36.00) | 14.95(65.00) | 3.90(2.23) |
| Cardiac Output Decreased | 5 | 65.09(27.00-156.92) | 65.03(313.17) | 6.01(4.34) |
| Miosis | 5 | 7.96(3.31-19.13) | 7.95(30.36) | 2.99(1.32) |
| Product Name Confusion | 5 | 44.28(18.39-106.64) | 44.24(210.35) | 5.46(3.79) |
| Motor Dysfunction | 5 | 5.90(2.45-14.18) | 5.89(20.31) | 2.56(0.89) |
| Supraventricular Extrasystoles | 5 | 16.39(6.81-39.42) | 16.37(72.05) | 4.03(2.36) |
| Injection Site Extravasation | 5 | 4.17(1.73-10.02) | 4.16(12.02) | 2.06(0.39) |
| Blood Pressure Systolic Decreased | 5 | 13.08(5.44-31.45) | 13.07(55.64) | 3.71(2.04) |
| Sinus Node Dysfunction | 5 | 18.84(7.83-45.32) | 18.82(84.21) | 4.23(2.56) |
| Mean Arterial Pressure Decreased | 5 | 245.08(100.86-595.53) | 244.85(1184.65) | 7.90(6.22) |
| Transcranial Electrical Motor Evoked Potential Monitoring Abnormal | 5 | 2723.16(1010.65-7337.50) | 2720.53(10637.74) | 11.06(9.29) |
| Blood Pressure Diastolic Decreased | 5 | 7.77(3.23-18.68) | 7.76(29.43) | 2.96(1.29) |
| Stridor | 5 | 19.94(8.29-47.97) | 19.92(89.69) | 4.31(2.65) |
| Hypercapnia | 5 | 20.10(8.35-48.35) | 20.08(90.46) | 4.32(2.66) |
| Sinus Arrhythmia | 5 | 42.07(17.47-101.32) | 42.03(199.44) | 5.39(3.72) |
| Paradoxical Drug Reaction | 5 | 16.94(7.04-40.75) | 16.93(74.80) | 4.08(2.41) |
| Respiratory Rate Decreased | 5 | 17.95(7.46-43.19) | 17.94(79.82) | 4.16(2.50) |
| Nephrogenic Diabetes Insipidus | 5 | 51.49(21.37-124.04) | 51.44(246.00) | 5.68(4.01) |
| Sinus Tachycardia | 5 | 4.07(1.69-9.78) | 4.07(11.56) | 2.02(0.36) |
| Product Appearance Confusion | 5 | 68.75(28.51-165.75) | 68.68(331.16) | 6.09(4.42) |
| Acidosis | 5 | 7.55(3.14-18.14) | 7.54(28.34) | 2.91(1.25) |
| Atrioventricular Block First Degree | 5 | 12.44(5.17-29.92) | 12.43(52.48) | 3.63(1.97) |
| Colitis Ischaemic | 5 | 8.85(3.68-21.27) | 8.84(34.73) | 3.14(1.48) |
| Respiration Abnormal | 5 | 9.31(3.87-22.40) | 9.31(37.04) | 3.22(1.55) |
| Recurrence Of Neuromuscular Blockade | 5 | 379.98(155.45-928.79) | 379.61(1817.61) | 8.51(6.83) |
| Haemophagocytic Lymphohistiocytosis | 4 | 5.53(2.07-14.74) | 5.52(14.82) | 2.47(0.80) |
| Hyperventilation | 4 | 7.75(2.90-20.65) | 7.74(23.46) | 2.95(1.28) |
| Bundle Branch Block Right | 4 | 9.54(3.58-25.43) | 9.53(30.51) | 3.25(1.58) |
| Hypoventilation | 4 | 13.33(5.00-35.54) | 13.32(45.51) | 3.73(2.07) |
| Increased Bronchial Secretion | 4 | 22.73(8.52-60.65) | 22.71(82.83) | 4.50(2.83) |
| Arrhythmia Supraventricular | 4 | 39.56(14.81-105.66) | 39.53(149.63) | 5.30(3.63) |
| Apgar Score Low | 4 | 33.54(12.56-89.54) | 33.51(125.74) | 5.06(3.39) |
| Sinoatrial Block | 4 | 71.02(26.55-189.99) | 70.97(273.96) | 6.14(4.47) |
| Cardiac Arrest Neonatal | 4 | 350.05(129.07-949.37) | 349.78(1343.17) | 8.40(6.71) |
| Stupor | 4 | 13.58(5.09-36.22) | 13.57(46.51) | 3.76(2.09) |
| Electrocardiogram Pr Prolongation | 4 | 56.25(21.04-150.35) | 56.21(215.66) | 5.80(4.13) |
| Lymphocyte Percentage Increased | 4 | 118.81(44.31-318.55) | 118.71(461.30) | 6.87(5.20) |
| Hypotonia | 4 | 4.53(1.70-12.07) | 4.53(10.99) | 2.18(0.51) |
| Cardiac Failure Acute | 4 | 7.53(2.82-20.07) | 7.52(22.61) | 2.91(1.24) |
| Brain Injury | 4 | 4.48(1.68-11.93) | 4.47(10.78) | 2.16(0.49) |
| Macule | 4 | 16.86(6.32-44.98) | 16.85(59.54) | 4.07(2.40) |
| Rhythm Idioventricular | 4 | 149.64(55.72-401.84) | 149.53(581.26) | 7.20(5.53) |
| Drug Withdrawal Syndrome Neonatal | 4 | 3.40(1.28-9.06) | 3.40(6.77) | 1.76(0.10) |
| Pulse Absent | 4 | 9.33(3.50-24.87) | 9.32(29.69) | 3.22(1.55) |
| Weaning Failure | 3 | 84.00(26.95-261.79) | 83.95(243.79) | 6.38(4.71) |
| Drug Tolerance | 3 | 7.02(2.26-21.78) | 7.02(15.46) | 2.81(1.14) |
| Anuria | 3 | 3.85(1.24-11.95) | 3.85(6.32) | 1.94(0.28) |
| Intercepted Product Dispensing Error | 3 | 13.03(4.20-40.45) | 13.02(33.26) | 3.70(2.03) |
| Ileus Paralytic | 3 | 8.09(2.61-25.10) | 8.08(18.61) | 3.01(1.35) |
| Laryngeal Oedema | 3 | 5.69(1.83-17.65) | 5.69(11.58) | 2.51(0.84) |
| Product Closure Issue | 3 | 79.24(25.43-246.90) | 79.20(229.77) | 6.30(4.62) |
| Electrocardiogram Repolarisation Abnormality | 3 | 41.70(13.41-129.65) | 41.68(118.60) | 5.38(3.71) |
| Long Qt Syndrome | 3 | 14.68(4.73-45.56) | 14.67(38.16) | 3.87(2.21) |
| Partial Seizures | 3 | 7.08(2.28-21.97) | 7.08(15.65) | 2.82(1.16) |
| Neonatal Hypoxia | 3 | 42.85(13.78-133.25) | 42.83(122.03) | 5.41(3.74) |
| Foetal Exposure During Delivery | 3 | 53.35(17.15-166.00) | 53.32(153.20) | 5.73(4.06) |
| Low Birth Weight Baby | 3 | 4.47(1.44-13.85) | 4.46(8.06) | 2.16(0.49) |
| Thyrotoxic Crisis | 3 | 38.48(12.38-119.62) | 38.46(109.02) | 5.26(3.59) |
| Urine Output Increased | 3 | 12.98(4.18-40.29) | 12.97(33.11) | 3.70(2.03) |
| Product Selection Error | 3 | 33.68(10.84-104.65) | 33.66(94.73) | 5.07(3.40) |
| Large Intestine Perforation | 3 | 5.06(1.63-15.69) | 5.05(9.75) | 2.34(0.67) |
| Coronary Artery Stenosis | 3 | 6.39(2.06-19.84) | 6.39(13.63) | 2.68(1.01) |
| Anaesthetic Complication | 3 | 13.08(4.21-40.61) | 13.08(33.41) | 3.71(2.04) |
| Tachyphylaxis | 3 | 97.03(31.11-302.62) | 96.97(282.15) | 6.59(4.91) |
| Neonatal Respiratory Depression | 3 | 104.25(33.41-325.30) | 104.19(303.37) | 6.69(5.01) |
| Atrioventricular Dissociation | 3 | 123.01(39.38-384.23) | 122.94(358.34) | 6.92(5.25) |
| Therapy Partial Responder | 3 | 3.93(1.27-12.18) | 3.93(6.54) | 1.97(0.31) |
| Postresuscitation Encephalopathy | 3 | 498.28(156.15-1590.03) | 498.00(1415.99) | 8.89(7.18) |
| Endotracheal Intubation | 3 | 11.95(3.85-37.09) | 11.94(30.05) | 3.58(1.91) |
| Brugada Syndrome | 3 | 37.12(11.94-115.38) | 37.10(104.98) | 5.21(3.54) |
| Intensive Care Unit Acquired Weakness | 3 | 75.97(24.38-236.65) | 75.92(220.10) | 6.24(4.56) |
| Epidermolysis Bullosa | 3 | 134.24(42.95-419.59) | 134.16(391.16) | 7.05(5.37) |
| Bradypnoea | 3 | 15.26(4.92-47.38) | 15.26(39.90) | 3.93(2.26) |
| Klebsiella Test Positive | 3 | 56.75(18.24-176.61) | 56.72(163.27) | 5.82(4.15) |
| Cerebral Artery Occlusion | 3 | 31.18(10.03-96.87) | 31.16(87.29) | 4.96(3.29) |
| Catatonia | 3 | 7.16(2.31-22.22) | 7.16(15.88) | 2.84(1.17) |
| Mechanical Ventilation Complication | 3 | 299.99(95.07-946.58) | 299.81(866.91) | 8.18(6.49) |
| Central Venous Pressure Increased | 3 | 277.35(88.01-874.02) | 277.19(802.85) | 8.07(6.39) |
| Staring | 3 | 14.05(4.52-43.60) | 14.04(36.28) | 3.81(2.14) |
| Salivary Hypersecretion | 3 | 3.60(1.16-11.17) | 3.60(5.63) | 1.85(0.18) |

PT = preferred term of the Medical Dictionary for Regulatory Activities

PRR = proportional reporting ratio

ROR = reporting odds ratio

IC = information component.
