# Supplementary material for: Dexmedetomidine: a real-world safety analysis based on FDA adverse event reporting system database
Source: Front Pharmacol. 2024 Aug 23;15:1419196. doi: 10.3389/fphar.2024.1419196 (PMC11377849; doi:10.3389/fphar.2024.1419196)
Supplement: Supplementary file 2 [file Table2.docx]

**Supplementary Table 2. New signals of Dexmedetomidine adverse events at the preferred terms level in FAERS database.**

| **PT** | **Case Reports** | **ROR(95%CI)** | **PRR(X2)** | **IC(IC025)** |
| --- | --- | --- | --- | --- |
| Transcranial Electrical Motor Evoked Potential Monitoring Abnormal | 5 | 2723.16 (1010.65-7337.50) | 2720.53 (10637.74) | 11.06 (9.29) |
| Acute Motor Axonal Neuropathy | 10 | 1509.67 (775.35-2939.47) | 1506.76 (13041.24) | 10.35 (8.65) |
| Trigemino-Cardiac Reflex | 7 | 1204.39 (549.10-2641.72) | 1202.76 (7486.01) | 10.07 (8.36) |
| Glossoptosis | 19 | 1079.59 (671.73-1735.12) | 1075.63 (18380.44) | 9.92 (8.24) |
| Floppy Iris Syndrome | 21 | 623.90 (401.04-970.58) | 621.37 (12230.84) | 9.19 (7.52) |
| Phaeochromocytoma Crisis | 7 | 524.05 (244.87-1121.49) | 523.34 (3464.28) | 8.96 (7.27) |
| Postresuscitation Encephalopathy | 3 | 498.28 (156.15-1590.03) | 498.00 (1415.99) | 8.89 (7.18) |
| Diabetes Insipidus | 75 | 389.00 (308.35-490.74) | 383.37 (27526.81) | 8.53 (6.86) |
| Recurrence Of Neuromuscular Blockade | 5 | 379.98 (155.45-928.79) | 379.61 (1817.61) | 8.51 (6.83) |
| Intestinal Pseudo-Obstruction | 29 | 347.47 (239.69-503.72) | 345.53 (9623.04) | 8.38 (6.71) |
| Hypocapnia | 6 | 170.53 (76.05-382.39) | 170.33 (992.75) | 7.39 (5.71) |
| Hyperthermia Malignant | 19 | 156.03 (99.09-245.69) | 155.46 (2870.35) | 7.26 (5.59) |
| Drug Withdrawal Convulsions | 22 | 141.06 (92.52-215.06) | 140.46 (3003.30) | 7.11 (5.45) |
| Epidermolysis Bullosa | 3 | 134.24 (42.95-419.59) | 134.16 (391.16) | 7.05 (5.37) |
| Lymphocyte Percentage Increased | 4 | 118.81 (44.31-318.55) | 118.71 (461.30) | 6.87 (5.20) |
| Intensive Care Unit Acquired Weakness | 3 | 75.97 (24.38-236.65) | 75.92 (220.10) | 6.24 (4.56) |
| Thyrotoxic Crisis | 3 | 38.48 (12.38-119.62) | 38.46 (109.02) | 5.26 (3.59) |
| Brugada Syndrome | 3 | 37.12 (11.94-115.38) | 37.10 (104.98) | 5.21 (3.54) |
| Cerebral Artery Occlusion | 3 | 31.18 (10.03-96.87) | 31.16 (87.29) | 4.96 (3.29) |
| Increased Bronchial Secretion | 4 | 22.73 (8.52-60.65) | 22.71 (82.83) | 4.50 (2.83) |
| Cardiac Tamponade | 9 | 22.26 (11.57-42.83) | 22.22 (181.98) | 4.47 (2.80) |
| Tachypnoea | 24 | 21.80 (14.59-32.57) | 21.70 (473.01) | 4.44 (2.77) |
| Hypoxic-Ischaemic Encephalopathy | 5 | 14.97 (6.22-36.00) | 14.95 (65.00) | 3.90 (2.23) |
| Staring | 3 | 14.05 (4.52-43.60) | 14.04 (36.28) | 3.81 (2.14) |
| Neuroleptic Malignant Syndrome | 14 | 13.80 (8.16-23.32) | 13.76 (165.51) | 3.78 (2.11) |
| Stupor | 4 | 13.58 (5.09-36.22) | 13.57 (46.51) | 3.76 (2.09) |
| Blood Albumin Decreased | 9 | 13.01 (6.76-25.03) | 12.99 (99.49) | 3.70 (2.03) |
| Prothrombin Time Prolonged | 7 | 12.14 (5.78-25.49) | 12.12 (71.36) | 3.60 (1.93) |
| Posterior Reversible Encephalopathy Syndrome | 10 | 12.10 (6.51-22.52) | 12.08 (101.53) | 3.59 (1.93) |
| Hiccups | 8 | 11.97 (5.98-23.96) | 11.95 (80.21) | 3.58 (1.91) |
| Anaphylactic Shock | 23 | 11.26 (7.47-16.96) | 11.21 (213.72) | 3.49 (1.82) |
| Atelectasis | 9 | 10.40 (5.41-20.01) | 10.39 (76.28) | 3.38 (1.71) |
| Hypoalbuminaemia | 6 | 9.61 (4.31-21.40) | 9.60 (46.17) | 3.26 (1.59) |
| Colitis Ischaemic | 5 | 8.85 (3.68-21.27) | 8.84 (34.73) | 3.14 (1.48) |
| Rhabdomyolysis | 30 | 8.54 (5.96-12.23) | 8.50 (198.40) | 3.09 (1.42) |
| Ileus Paralytic | 3 | 8.09 (2.61-25.10) | 8.08 (18.61) | 3.01 (1.35) |
| Miosis | 5 | 7.96 (3.31-19.13) | 7.95 (30.36) | 2.99 (1.32) |
| Hyperventilation | 4 | 7.75 (2.90-20.65) | 7.74 (23.46) | 2.95 (1.28) |
| Catatonia | 3 | 7.16 (2.31-22.22) | 7.16 (15.88) | 2.84 (1.17) |
| Restless Legs Syndrome | 11 | 6.97 (3.86-12.60) | 6.96 (56.13) | 2.80 (1.13) |
| Ileus | 7 | 6.94 (3.30-14.56) | 6.93 (35.49) | 2.79 (1.13) |
| Multiple Organ Dysfunction Syndrome | 26 | 6.87 (4.67-10.10) | 6.84 (129.61) | 2.77 (1.11) |
| Motor Dysfunction | 5 | 5.90 (2.45-14.18) | 5.89 (20.31) | 2.56 (0.89) |
| Hypocalcaemia | 9 | 5.74 (2.99-11.04) | 5.73 (35.16) | 2.52 (0.85) |
| Laryngeal Oedema | 3 | 5.69 (1.83-17.65) | 5.69 (11.58) | 2.51 (0.84) |
| Haemophagocytic Lymphohistiocytosis | 4 | 5.53 (2.07-14.74) | 5.52 (14.82) | 2.47 (0.80) |
| Cerebral Infarction | 11 | 5.14 (2.84-9.29) | 5.13 (36.58) | 2.36 (0.69) |
| Large Intestine Perforation | 3 | 5.06 (1.63-15.69) | 5.05 (9.75) | 2.34 (0.67) |
| Hypotonia | 4 | 4.53 (1.70-12.07) | 4.53 (10.99) | 2.18 (0.51) |
| Brain Injury | 4 | 4.48 (1.68-11.93) | 4.47 (10.78) | 2.16 (0.49) |
| Low Birth Weight Baby | 3 | 4.47 (1.44-13.85) | 4.46 (8.06) | 2.16 (0.49) |
| Premature Delivery | 6 | 4.34 (1.95-9.67) | 4.34 (15.39) | 2.12 (0.45) |
| Salivary Hypersecretion | 3 | 3.60 (1.16-11.17) | 3.60 (5.63) | 1.85 (0.18) |
| Encephalopathy | 7 | 3.43 (1.63-7.19) | 3.42 (12.01) | 1.78 (0.11) |
| Dyskinesia | 12 | 3.42 (1.94-6.03) | 3.42 (20.53) | 1.77 (0.11) |
| Premature Baby | 9 | 3.42 (1.78-6.58) | 3.41 (15.37) | 1.77 (0.10) |
| Haematocrit Decreased | 6 | 3.30 (1.48-7.35) | 3.30 (9.60) | 1.72 (0.05) |

PT = preferred term of the Medical Dictionary for Regulatory Activities

PRR = proportional reporting ratio

ROR = reporting odds ratio

IC = information component.
